# Water-soluble cranberry extract inhibits *Vibrio cholerae* biofilm formation possibly through modulating the second messenger 3’, 5’ - Cyclic diguanylate level

**DOI:** 10.1371/journal.pone.0207056

**Published:** 2018-11-07

**Authors:** Daniel B. Pederson, Yuqing Dong, Levi B. Blue, Sara V. Smith, Min Cao

**Affiliations:** 1 Department of Biological Sciences, Clemson University, Clemson, South Carolina, United States of America; 2 Institute for Engaged Aging, Clemson University, Clemson, South Carolina, United States of America; Purdue University, UNITED STATES

## Abstract

Quorum sensing (QS) and nucleotide-based second messengers are vital signaling systems that regulate bacterial physiology in response to changing environments. Disrupting bacterial signal transduction is a promising direction to combat infectious diseases, and QS and the second messengers are undoubtedly potential targets. In *Vibrio cholerae*, both QS and the second messenger 3’, 5’—cyclic diguanylate (c-di-GMP) play a central role in controlling motility, motile-to-sessile life transition, and virulence. In this study, we found that water-soluble extract from the North American cranberry could significantly inhibit *V*. *cholerae* biofilm formation during the development/maturation stage by reducing the biofilm matrix production and secretion. The anti-biofilm effect by water-soluble cranberry extract was possibly through modulating the intracellular c-di-GMP level and was independent of QS and the QS master regulator HapR. Our results suggest an opportunity to explore more functional foods to fight stubborn infections through interference with the bacterial signaling systems.

## Introduction

Quorum sensing (QS) and the nucleotide-based second messengers, especially the cyclic dinucleotides, are two central signaling systems utilized by many bacteria to regulate their physiological functions in response to changing environmental conditions or during the developmental process. Due to their decisive roles in bacterial physiology, QS and the second messengers have been considered as potential targets for new drug development to tackle the increasingly grim situation of antibiotic resistance. By blocking the signaling transduction rather than targeting the essential genes, placing selective pressure on resistant strains of bacteria is avoided. In the past twenty years, natural QS inhibitors (QSIs) have been identified from a number of organisms, and a list of synthetic QSIs have also been developed in research labs [1, 2]. In contrast, finding inhibitors of the cyclic dinucleotide-based signaling pathways has progressed slowly. To date, only a very limited number of compounds have been characterized as cyclic dinucleotide signaling inhibitors [3].

In the Gram-negative bacterial pathogen *Vibrio cholerae*, both QS and the cyclic dinucleotides play a fundamental role in controlling cell motility, biofilm formation, and virulence gene expression. *V*. *cholerae* is the causative agent of a frequently fatal disease called cholera. Since the first cholera pandemic occurred ~ 200 years ago, the disease has affected millions of people. With a better knowledge of the disease control and improved water and sanitation facilities, the disease transmission has been eliminated in the developed countries, yet cholera remains a threat in many parts of the developing world. An essential component to this pathogen’s success and persistence in the environment is its ability to attach to both biotic and abiotic surfaces via biofilm formation [4]. Biofilms not only aid in surface attachment, they also provide a barrier that protects and enhances survival. In human infection, when *V*. *cholerae* enters the body, it must first survive the acidic environment of the stomach and then proceed to attach to the intestinal wall. Biofilms provide cells resistance to high acidity and therefore are critical for the transmission and infectivity of *V*. *cholera*e [5, 6]. The major component of *V*. *cholerae* biofilm, Vibrio polysaccharide, is synthesized by enzymes encoded in the two *vps* (Vibrio polysaccharide synthesis) gene operons (*vps*-I and *vps*-II) [7, 8]. Additionally, genes from the intergenic region of the two operons (*rbmA*, *rbmB*, and *rbmC*) as well as *bap1*, which is located downstream of *vps*-II, are involved in the production of matrix proteins and maintenance of the biofilm structure. RbmA provides cell-cell adhesion, RbmC and Bap1 form the envelopes to encase the cell clusters, and RbmB, whose function is yet to be characterized, is proposed to help with the detachment of bacteria from mature biofilms [9–11]. The two *vps* operons are positively regulated by two major transcriptional regulators, VpsR and VpsT [12, 13], and *rbmA*, *rbmB*, *rbmC*, and *bap1* are positively regulated by VpsR [11, 14]. Expression of *vpsR* and *vpsT* is regulated by the cell density through the upstream QS pathway, and by the intracellular concentration of 3’, 5’—cyclic diguanylate (c-di-GMP), an important second messenger identified in a wide variety of bacteria [15–17].

Unlike many other pathogenic bacteria that cause persistent infections in which QS typically activates biofilm formation and virulence at high cell density, in *V*. *cholerae*, genes involved in biofilm formation and virulence are maximally expressed at low cell density and are turned off at high cell density. This unique system is thought to aid in *V*. *cholerae*’s life cycle during acute infection, allowing it to colonize in the intestine and promoting infection at low cell density, and helping the bacteria detach and transition back to its natural environment at high cell density [5, 18, 19]. *V*. *cholerae* responds to at least two QS signaling molecules (called autoinducers), CAI-1 and AI-2, through the response regulator LuxO. At low cell density, LuxO is in the phosphorylated form and activates expression of a set of small regulatory RNAs, which in turn inhibits expression of the major QS regulator HapR, allowing expression of *vpsR*, *vpsT*, as well as virulence genes. At high cell density, LuxO is dephosphorylated; thus HapR is de-repressed and inhibits biofilm and virulence genes.

In *V*. *cholerae*, increasing the intracellular c-di-GMP level promotes the transition from motile to sessile lifestyle [20–22]. C-di-GMP has been shown to regulate both the initial attachment and biofilm matrix formation. Binding of c-di-GMP to the ATPase MshE promotes the polymerization of the MshA subunits to form the pili, which are critical for initial surface attachment [23]. Both the transcription activators VpsR and VpsT can sense c-di-GMP and directly bind to it, leading to activation of biofilm genes [24, 25]. C-di-GMP is synthesized by the diguanylate cyclases (DGCs) containing a GGDEF domain and degraded by the phosphodiesterases (PDEs) containing either an EAL or HD-GYP domain [26]. The *V*. *cholerae* genome encodes 31 proteins with the GGDEF domain, 12 proteins with the EAL domain, nine proteins with the HD-GYP domain, and ten proteins with both GGDEF and EAL domains [27, 28]. Prior research has identified a list of DGCs and PDEs involved in regulating *V*. *cholerae* c-di-GMP level and biofilm formation [21, 29, 30]. Many DGCs and PDEs have N-terminal sensory input domain, indicating their activities could be modulated by various environmental signals, which in turn affects the intracellular c-di-GMP level [28].

Previously, we reported that supplementation of water-soluble cranberry extract standardized to 4.0% proanthocyanidins (WCESP) to *Caenorhabditis elegans*, a nematode model, could significantly enhance the host’s resistance to infection by various bacterial pathogens including *Vibrio cholerae*. Mechanistic studies showed that the WCESP-mediated protection was mainly through promoting the host’s innate immunity. Under our experimental condition, WCESP treatment did not affect the growth of *V*. *cholerae* or expression of the major bacterial virulence genes, but a slight reduction of bacterial colonization within *C*. *elegans* intestine was noticed [31]. In this study, we tested WCESP’s ability to inhibit *V*. *cholerea* biofilm formation and further investigated the inhibition mechanism. We found that low concentration of WCESP could significantly impede *V*. *cholerae* biofilm formation during the development/maturation stage; this inhibition is independent of the QS pathway but is possibly through modulating the intracellular c-di-GMP level. This study suggests that cranberry contains certain active constituents that can interfere with *V*. *cholerae* c-di-GMP signal transduction and provides a potential means to control *V*. *cholerae* biofilm.

## Material and methods

### Plasmids, bacterial strains, and growth media

The plasmid pBAD33 was obtained from Coli Genetic Stock center (CGSC), and pAT1662 (pBAD33::VCA0956-His_6_) was obtained from Dr. Andrew Camilli (Tufts University) [32]. The *V*. *cholerae* strains (except the MO10 strain) used in this study were all derived from the wild-type C6706 strain (O1 serotype El Tor isolated from Peru) [33]. The C6706 with P_*hapR*_-*lacZ* fusion, *luxO*^*-*^ (*luxO* deletion), *hapR*^*-*^ (*hapR* deletion), *luxO*^*C*^ (constitutively active LuxO), *cqsA*^*-*^ (*cqsA* deletion), *luxS*^*-*^ (*luxS* deletion), *cqsA*^*-*^*/luxS*^*-*^ (*cqsA* and *luxS* double deletion) strains were obtained from Dr. Jay Zhu (University of Pennsylvania) [5, 19, 34]. The MO10 strain (serotype O139) was isolated during the cholera outbreak in India and Bangladesh in 1992 [35, 36], also obtained from Dr. Jay Zhu. Other bacterial strains used in this study were *Staphylococcus aureus* (*S*. *aureus*, ATCC^#^25923), *Pseudomonas aeruginosa* (*P*. *aeruginosa*, ATCC^#^27853), *Enterococcus faecalis* (*E*. *faecalis*, ATCC^#^47077), *Salmonella typhimurium* (*S*. *typhimurium*, ATCC^#^14028), *E*. *coli O157*:*H7* (ATCC^#^700927), *Listeria monocytogenes* (*L*. *monocytogenes*) ScottA (serotype 4b), and *L*. *monocytogenes* Mac (serotype 1/2a). The *E*. *coli*, *V*. *cholerae*, *P*. *aeruginosa*, and *S*. *typhimurium* strains were cultured in Lysogeny broth (LB). The *S*. *aureus*, *E*. *faecalis*, and *L*. *monocytogenes* strains were cultured in brain-heart infusion (BHI) medium.

### Preparation of water-soluble cranberry extract

The cranberry extract used in this study was obtained from Naturex-DBS, LLC (Sagamore, MA, USA) as described in previously published studies [31]. A stock solution of WCESP was freshly prepared by dissolving the powder in distilled water to a concentration of 50 mg/ml immediately before use.

### Biofilm growth and quantification

Overnight bacterial cultures were inoculated (1:100 dilution) into 1 ml of LB or BHI medium in the presence or absence of 2 mg/ml of WCESP and incubated statically in polystyrene tubes for 24 hours to allow biofilm development. The temperatures used to develop biofilms were 25°C for *V*. *cholerae*, and 37°C for other bacterial strains. Quantifications of sessile/biofilm cells were conducted by the crystal violet staining method and the direct enumeration of live cells. For the staining method, the planktonic bacterial cells were carefully removed from each tube, and the tube was washed twice with PBS. The sessile bacterial cells were stained with 1% crystal violet for 30 minutes, followed by distilled water wash for five times. To enumerate biofilm bacterial cells, planktonic cells were carefully removed from underneath the biofilm pellicle. The pellicle and surface-attached cells were washed twice with PBS and resuspended in 1 ml of PBS. The cells were vortexed with glass beads (ϕ = 1mm) for two minutes to disrupt the biofilm and then plated onto LB agar plates with proper dilutions. Colonies were counted on the second day. In a parallel set of experiments, total bacterial cells (both planktonic and biofilm cells) were measured by first vortexing the cells with glass beads and then plating onto LB agar plates with proper dilutions. Data shown are the average results from three independent experiments, and triplicate samples were tested each time. The data were pooled and analyzed using unpaired Student’s t-test. A *p*-value < 0.05 was accepted as statistically significant.

### Biofilm inhibition in pre-established *V*. *cholerae* culture

The overnight culture of *V*. *cholerae* was inoculated into 1ml of LB in polystyrene test tubes (1:100 dilution) and incubated statically at 25°C for 4 hours. Then, 40 μl of WCESP stock solution (50 mg/ml) was added to the test tubes to reach a final concentration of 2 mg/ml. In the control tubes, 40 μl of sterile ddH_2_O was added. The tubes were incubated at 25°C under static condition for the remaining 20 hours. Biofilm cells were measured using the standard plate count method as described above. Data shown are the average results from three independent experiments, and triplicate samples were tested each time. The data were pooled and analyzed using unpaired Student’s t-test. A *p*-value < 0.05 was accepted as statistically significant.

### Motility assay

Overnight cultures of *V*. *cholerae* were inoculated into 1 ml of LB with or without 2 mg/ml WCESP in polystyrene test tubes (1:100 dilution). Cultures were incubated with shaking at 150 rpm at 25°C until reaching mid-log phase. Three microliters of the culture were dropped onto motility assay plates (LB with 0.3% agar). The plates were incubated at 25°C. The diameters of the motility zones were measured after 4 and 24 hours, respectively.

### Cell-surface hydrophobicity measurement

The cell-surface hydrophobicity was measured based on the method described by Prabu et al. [37]. Overnight *V*. *cholerae* C6706 culture was inoculated (1:100 dilution) into LB with or without 2 mg/ml of WCESP and incubated at 25°C with gentle shaking. Cell samples were collected at 4 and 24 hours respectively and washed twice with 0.85% NaCl. Samples were then transferred to glass test tubes and resuspended in 3 ml of 0.85% NaCl. Spectrophotometer reading was then taken from each sample at 600nm and recorded as the *OD Initial*. Next, 0.25 ml of toluene was added to each sample and vortexed for 2 minutes. Samples were then allowed to equilibrate at room temperature for 30 minutes. Following the complete separation of the toluene phase from the aqueous phase, OD of the aqueous phase of each sample was measured at 600nm and recorded as the *OD Final*. The Hydrophobicity Index (HPBI) was then calculated using the following formula: HPBI = (OD Initial—OD Final) / OD Initial x 100%. Data shown are the average results from three independent experiments, and triplicate samples were tested each time. The data were pooled and analyzed using unpaired Student’s t-test. A *p*-value < 0.05 was accepted as statistically significant.

### β-galactosidase assay for P_*hapR*_-*lacZ*

The overnight culture of *V*. *cholerae* C6706/P_*hapR*_-*lacZ* was inoculated into LB medium (1:100 dilution) with or without 2 mg/ml WCESP. Cultures were incubated statically at 25°C, and 1 ml of planktonic bacterial sample was collected at 4, 8, 16, and 24 hours, respectively. The β-galactosidase activity of each sample was measured according to the method developed by Miller [38]. Data shown are the average results from three independent experiments, and triplicate samples were tested each time. The data were pooled and analyzed using unpaired Student’s t-test. A *p*-value < 0.05 was accepted as statistically significant.

### Gene expression analysis by quantitative real-time PCR (qRT-PCR)

Overnight *V*. *cholerae* C6706 culture was inoculated (1:100 dilution) into 1 ml of LB with or without 2 mg/ml WCESP. Cultures were grown statically at 25°C. Planktonic bacterial samples were harvested after 4 and 24 hours of incubation and washed once with PBS. Total RNA was prepared using RNAzol RT reagent (Molecular Research Center, INC.) and stored at -80°C. Complementary DNA was synthesized using the iScript cDNA synthesis kit (Bio-Rad). qRT-PCR was performed using SensiFAST SYBR No-Rox Kit (Bioline) and the CFX96 real-time PCR detection system according to the manufacturer’s suggested protocol (Bio-Rad). The qRT-PCR conditions were: 95°C for 2 minutes, followed by 40 cycles of 5 seconds at 95°C, 10 seconds at 50°C, and 15 seconds at 72°C. 16S rRNA was used as the endogenous control to normalize the expression levels of target transcripts [31]. Relative fold-changes for transcripts were calculated using the comparative CT (2^−ΔΔCT^) method [39]. Cycle thresholds of amplification were determined by Light Cycler software (Bio-Rad). Each qRT-PCR experiment was repeated three times using independent RNA preparations. The data were pooled and analyzed using unpaired Student’s t-test, and a *p*-value < 0.05 was accepted as statistically significant. The primers used in this study are listed in Table 1.

**Table 1:** Oligos used in qRT-PCR.

Oligo	Sequence
16S rRNA-F	5'-GGAAACGATGGCTAATACCG-3'
16S rRNA-R	5'-GCCCTTACCTCACCAACTAG-3'
bap1-F	5'-CGCTGGCACACTAAACAA G-3'
bap1-R	5'-CCATACATTCATACCCAAGAGC-3'
epsE-F	5'-CTAACCCAAGTCTATCAG-3'
epsE-R	5'-AATCTTCGTTTTGAGGC-3'
flaA-F	5'-GGATTAAAGATACGGATTTTG-3'
flaA-R	5'-CGAGATTGCAGAGTTTG-3'
hapR-F	5'-CGATTGTCACTGGCTCAAAG-3'
hapR-R	5'-GCAGTTGGTTAGTTCGGTTG-3'
luxO-F	5'-GCGAAAGTGGTACAGGTAAAG-3'
luxO-R	5'-CCCTTTGACGTGACCAAAC-3'
mshA-F	5'-CATTGCCCATAAGTTTG-3'
mshA-R	5'-GTTCCTGTAGACGATTG-3'
pomB-F	5'-CTCTTGCTCTCGTTTTC-3'
pomB-R	5'-AATACTGGTCCCTTTGG-3'
rbmA-F	5'-TGGGTTCCAGAGTATATG-3'
rbmA-R	5'-GAGTTCAGGTAGGCTATT-3'
rbmB-F	5'-CAGCAGGAACAGAAATG-3'
rbmB-R	5'-CCTTAGCTCCTCTAGTATC-3'
rbmC-F	5'-CGAGCAATAAGAAAGTGG-3'
rbmC-R	5'-GCCTTCAACTAACCAAC-3'
vpsD-F	5'-CATCCAAGAGCAACTGAAAG-3'
vpsD-R	5'-GCAAGGTCAACACATTACGAG-3'
vpsL-F	5'-TTCTTTACATACGGCATTC-3'
vpsL-R	5'-GCCAATAAAAGAACCGAC-3'
vpsR-F	5'-GGCCATGTATTGGTATTGTGG-3'
vpsR-R	5'-GGCAAATGGTATCTGAACTGAG-3'
vpsT-F	5'-GTCCGCAGGATATTGAGCAT-3'
vpsT-R	5'-GCCTTTGATCAGGGTATCCA-3'

### c-di-GMP quantification assay

The thiazole orange (TO)-based fluorescent detection method was used to quantify the intracellular c-di-GMP level [40]. In brief, overnight *V*. *cholerae* C6706 culture was inoculated (1:100 dilution) into LB medium and incubated statically at 25°C. The planktonic cells from the static cultures were collected at 4h, 8h, 16h, and 24h time-points to represent the key growth phases, and the total cell number in each sample was determined using the plate count method. Bacterial cells were pelleted by centrifugation and resuspended in 1 ml of 10 mM Tris-HCl (pH 8.0) buffer containing 100 mM NaCl. The cells were lysed by sonication. Trichloroacetic acid was added to the cell lysate to a final concentration of 12% to precipitate cellular macromolecules. The suspension was incubated on ice for 10 minutes and neutralized by 3 M KOH containing 0.4 M Tris and 2 M KCl, followed by centrifugation at full speed for 10 minutes. The supernatant was filtered through a 0.2 μm filter and then 1:10 diluted into the reaction buffer (10 mM Tris-HCl (pH 8.0) containing 1M NaCl), incubated at 95°C for 5 minutes, and cooled to room temp. TO was then added to each sample at a working concentration of 30 μM and incubated at 4°C for 12h until fluorescence readings were taken at excitation and emission wavelengths at 508nm and 557nm, respectively. The concentration of c-di-GMP was calculated using a calibration curve and normalized to 1 x 10^9^ bacterial cells in 1 ml.

## Results

### WCESP at non-lethal concentration specifically inhibits *V*. *cholerae* biofilm formation

**Fig 1:**
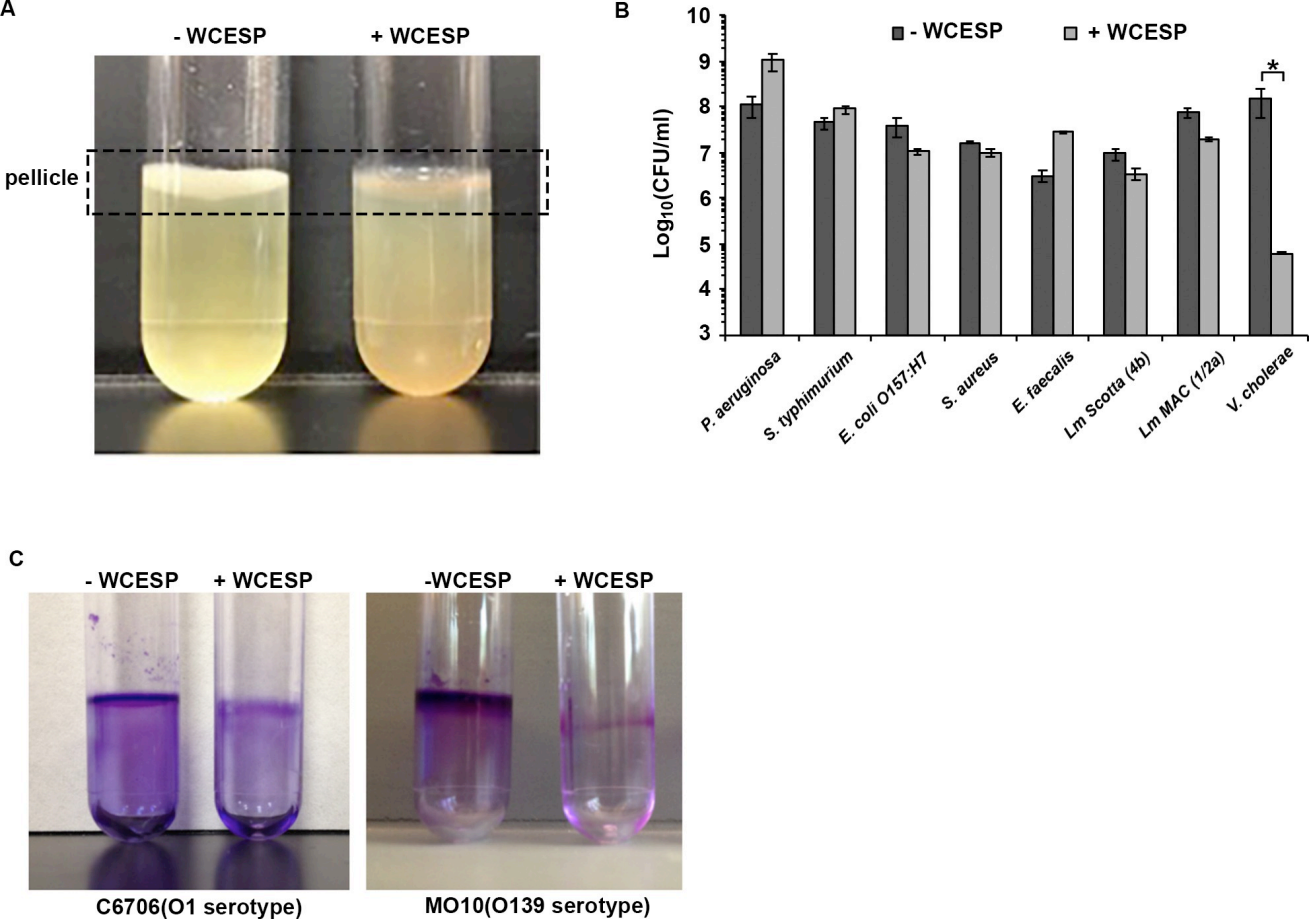
WCESP inhibits *V*. *cholerae* biofilm formation. The bacterial cultures were incubated for 24 hours without shaking in the absence and presence of 2 mg/ml of WCESP. (A) Biofilm pellicle formed at the air-water interface in the absence of WCESP, but not in the presence of WCESP. (B) Quantification of the pellicle and surface-attached bacterial cells of *V*. *cholerae* C6706 and other pathogenic bacteria in the presence and absence of WCESP. Results shown are the average of three independent experiments, and triplicate samples were tested each time; error bars are standard error of the mean. (* indicates *p* < 0.05). (C) Crystal violet staining results of surface-attached *V*. *cholerae* bacterial cells.

To test whether WCESP could affect *V*. *cholerae* biofilm formation, we grew *V*. *cholerae* wild-type C6706 strain statically in LB medium at 25°C, a temperature that more closely resembles the natural condition. WCESP was added into the growth medium at the same time during inoculation to reach a final concentration of 2 mg/ml. After incubation for 24 hours, the outcome of adding WCESP was palpable. As shown in Fig 1A, a thick pellicle was formed at the air-liquid interface in the control tube but not in the tube with WCESP. The bacterial culture in the WCESP tube also showed higher turbidity than that in the control tube, indicating that most of the bacterial cells remained in the planktonic form when WCESP was added into the medium. A standard plate count method was used to enumerate the bacterial cells in the pellicles and those attached to the wall of the culture tubes from both tubes, i.e., biofilm cells. An apparent reduction of cell numbers was observed in the WCESP tube (Fig 1B). In a parallel set of tubes, we quantified the total bacterial cells (both planktonic and biofilm cells) in the presence or absence of 2 mg/ml of WCESP by plate counting during the 24-hour time course. No visible difference in cell numbers was detected, indicating the WCESP concentration used in the biofilm inhibition assays did not cause any growth defects, which is consistent with our previous results (S1 Table) [31]. Considering that *V*. *cholerea* is a human pathogen, we also tested WCESP’s ability to inhibit biofilm at 37°C. A similar inhibition effect was observed as well (S1 Fig).

To test whether the biofilm inhibition by WCESP is a general feature towards a broad spectrum of bacterial pathogens, we examined seven other bacterial species including both Gram-positive and Gram-negative bacteria from our lab collections. No inhibition of biofilm formation by 2 mg/ml of WCESP was observed for any of the seven tested strains (Fig 1B). The wild-type *V*. *cholerae* strain used in our experiments is a clinical isolate belonging to the O1 serotype El Tor biotype. We further tested the WCESP’s impact on a *V*. *cholerae* MO10 strain, which belongs to the O139 serotype by a quick crystal violet staining method. Strong inhibition of biofilm formation by WCESP was detected (Fig 1C). Thus, our results revealed that at non-lethal concentration, WCESP specifically inhibits *V*. *cholerae* biofilm formation.

### WCESP impedes mainly the development/maturation stage of *V*. *cholerae* biofilm formation

**Fig 2:**
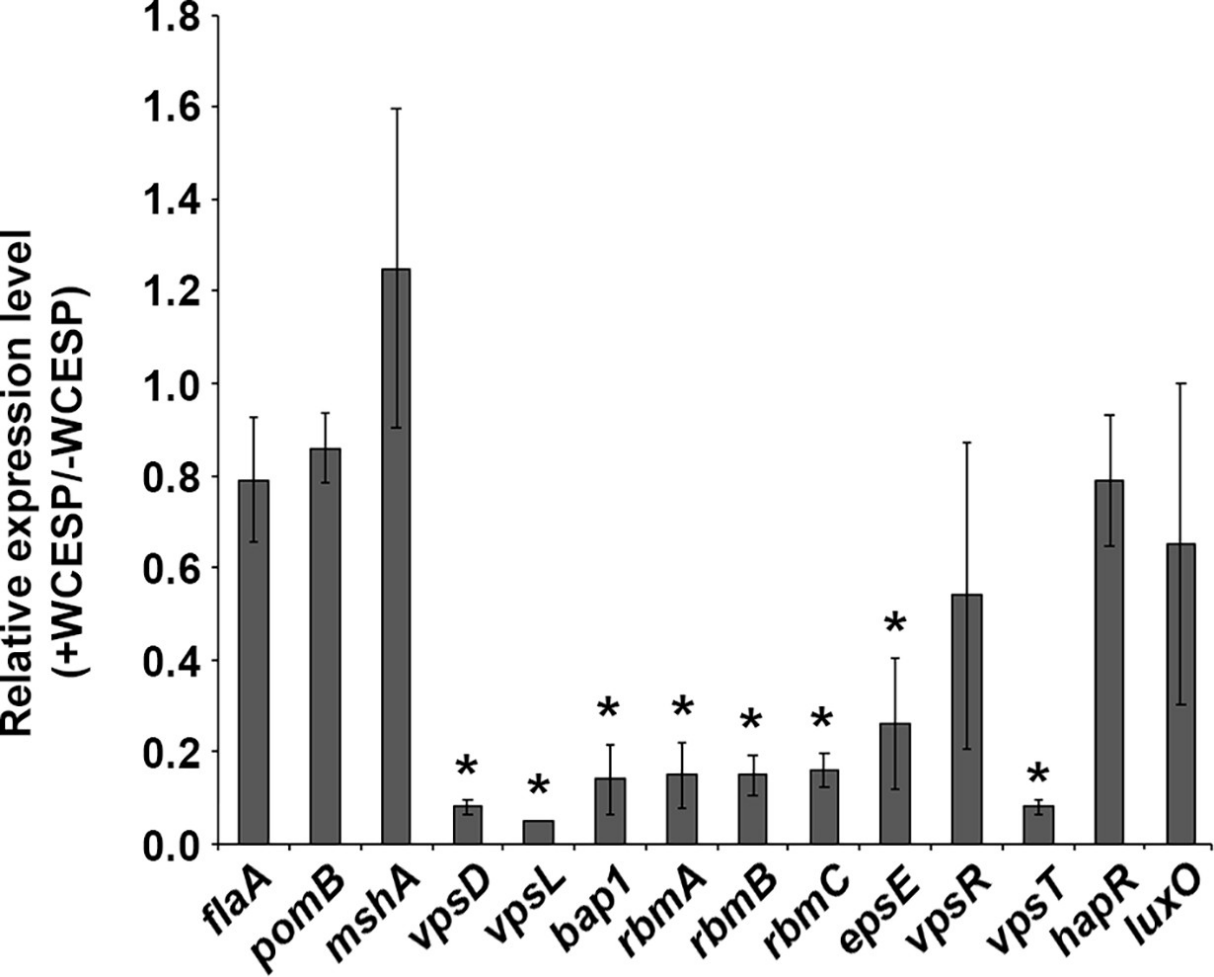
qRT-PCR analysis of *V*. *cholerae* biofilm-related genes in response to 2 mg/ml WCESP (normalized to 16S rRNA). Results are the average of three independent experiments, and error bars are standard error of the mean. (* indicates *p* < 0.05).

**Fig 3:**
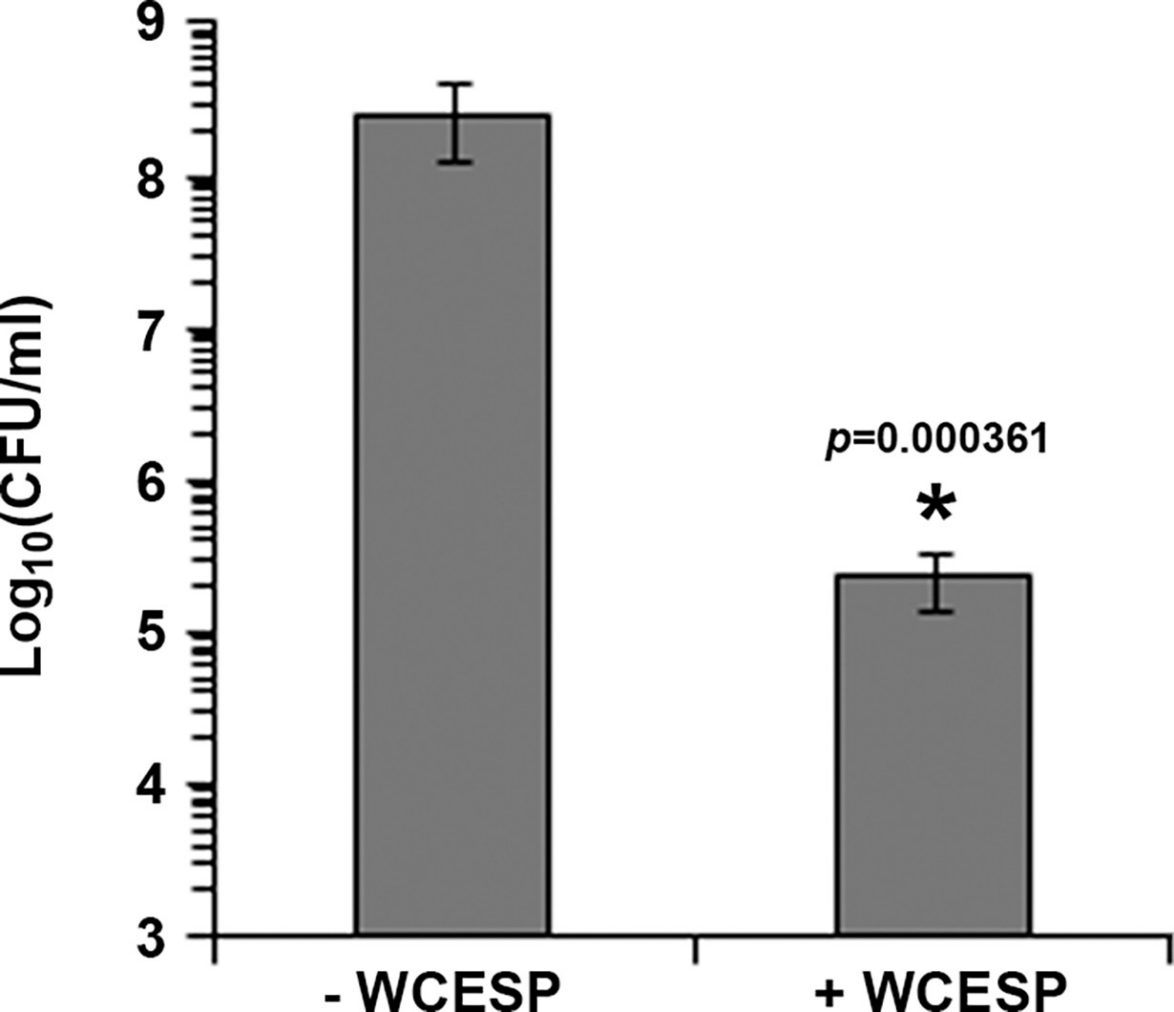
WCESP impedes mainly the development stage of *V*. *cholerae* biofilm formation. Inhibition of 4-hr pre-established biofilm formation by 2 mg/ml WCESP. Results shown are the average of three independent experiments, and triplicate samples were tested each time; error bars are standard error of the mean. (* indicates *p* < 0.05).

Biofilm formation is generally divided into initiation, development/maturation, and dispersion stages. Flagellar motility and the mannose-sensitive haemagglutinin type IV pilus (MSHA) are crucial for the initial attachment to abiotic surfaces, and the flagellum also mediates the spread across the surface area [41]. Mass production of exopolysaccharide (EPS) and matrix proteins is associated with the development of the three-dimensional structures during the maturation stage. To test which stage or stages were possibly affected by the addition of WCESP, we first performed a motility assay. The wild-type *V*. *cholerae* C6706 was grown to mid-log phase with shaking in the presence or absence of WCESP, and then 3 μl of the bacterial culture were dropped onto the motility agar plate and incubated at room temperature. The diameters of the motility zones were measured after 4 and 24 hours. As shown in Table 2, adding WCESP to the growth medium did not hinder flagellar motility. We further compared the expression of *flaA* (encoding the core flagellin), *pomB* (encoding the flagellar motor protein), and *mshA* (encoding the type IV pilus) in the presence or absence of WCESP using qRT-PCR. No noticeable changes were detected after either 4 hours (S2 Fig) or 24 hours of WCESP treatment (Fig 2). These results suggested that WCESP does not interfere with the initial attachment stage. We reasoned that if the initiation stage is not affected, we would see the same degree of inhibition if WCESP were added into a static culture that has already passed the first stage (we named it “pre-established culture”). As expected, the inhibition of a 4 hour-pre-established culture was evident and comparable to the result obtained when WCESP was added at inoculation (Fig 3). The same level of inhibition was also achieved on a 6 hour-pre-established culture. Together, these data indicated that WCESP impedes mainly the development/maturation stage of *V*. *cholerae* biofilm formation.

**Table 2:** The diameters of the motility zones.

	**T = 4 hr**	**T = 24 hr**
no WCESP	0.30 ± 0.00 cm	16.17 ± 0.29 cm
WCESP (2mg/ml) in the broth^a^	0.32 ± 0.03 cm	15.83 ± 0.29 cm
WCESP (2mg/ml) in the agar^b^	0.30 ± 0.00 cm	16.67 ± 0.29 cm

Note: The data shown are the average results from three repeats.

^a^ *V*. *cholerae* was cultured in the presence of WCESP until mid-log phase then dropped onto the motility plate.

^b^ *V*. *cholerae* was cultured in the absence of WCESP until mid-log phase then dropped onto the motility plate which contains WCESP.

### WCESP reduces *V*. *cholerae* cell-surface hydrophobicity, VPS, and major matrix proteins production and secretion

**Fig 4:**
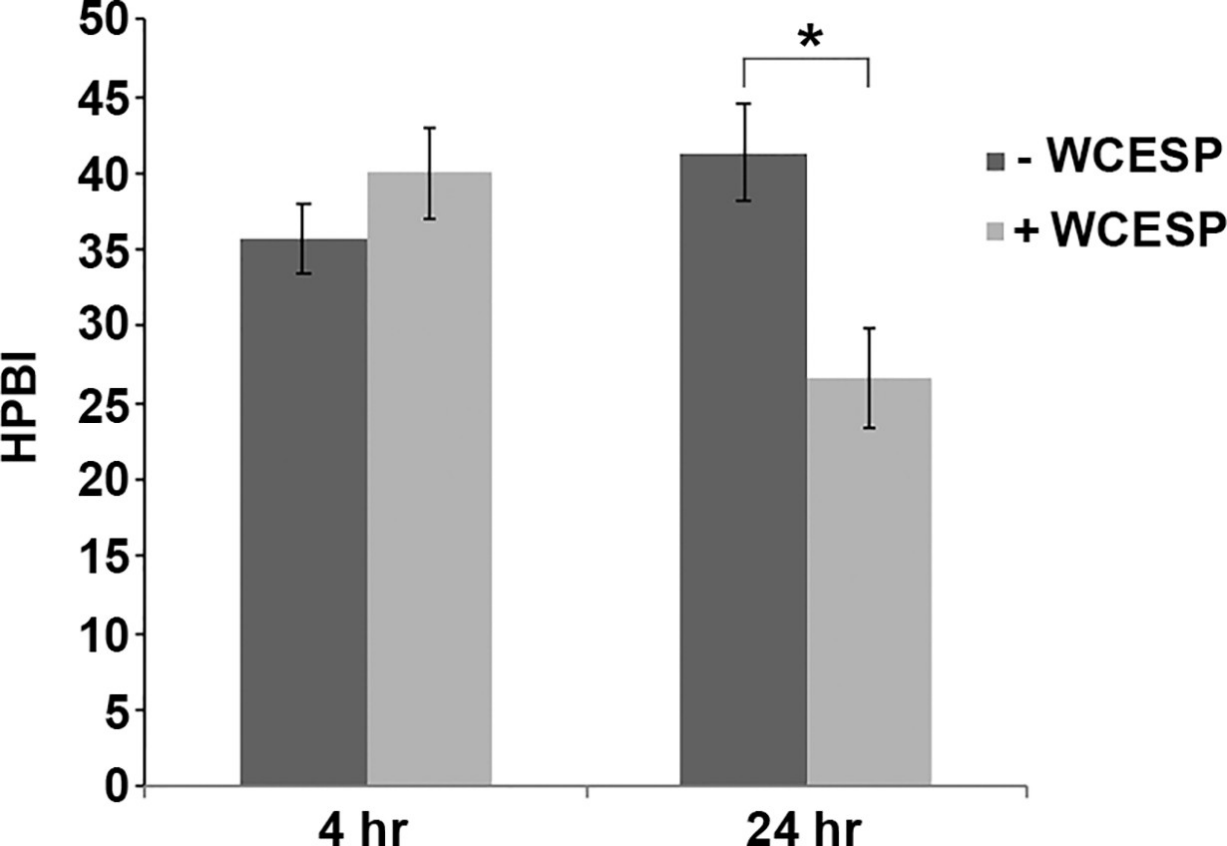
WCESP (2 mg/ml) decreases *V*. *cholerae* cell-surface hydrophobicity. The Hydrophobicity Index (HPBI) was calculated using the following formula: HPBI = (OD Initial—OD Final) / OD Initial x 100%. Data shown are the average results from three independent experiments, and triplicate samples were tested each time; error bars are standard error of the mean. (* indicates *p* < 0.05).

Bacterial cell-surface hydrophobicity (CSH) depicts the tendency of a bacterial cell to aggregate with cells of similar hydrophobicity as opposed to water, which is mainly affected by the bacterial cell surface composition and structure [42]. CSH also influences bacterial surface adhesion and biofilm development. Early study by Dahlbäck et al. showed that the hydrophobic interactions among bacteria played an important role in their initial adhesion at the air-water interface [43]. In *V*. *cholerae*, the biofilm matrix protein Bap1 is the crucial determinant of pellicle mechanical strength and hydrophobicity, allowing the pellicle to spread and remain at the air-liquid interface [44]. To test whether WCESP would change CSH, we incubated *V*. *cholerae* C6706 with and without 2 mg/ml WCESP and collected bacterial samples after 4 and 24 hours for the CSH measurement. As shown in Fig 4, there was no obvious difference of the hydrophobicity index (HPBI) between the 4-hour samples. But after 24 hours, the WCESP treated cells had a significant decrease of CSH (HPBI = 26.6 ± 3.3 vs. 41.3 ± 3.2 of untreated cells).

Using qRT-PCR, the expression of genes that are responsible for the production of VPS and matrix proteins was compared in the presence and absence of WCESP at 4 and 24 hours. No significant difference was detected at 4 hr. At 24 hr, downregulation of the *vpsD* (the fifth gene of the *vps-*I operon), *vpsL* (the first gene of the *vps-*II operon), as well as genes encoding the matrix proteins *bap1*, *rbmA*, *rbmB*, and *rbmC*, was evident in the presence of WCESP (Fig 2). Expression of the two transcriptional activators of the *vps* and *rbm* operons, VpsT and VpsR, was also measured. While *vpsT* expression showed a 12.5-fold decrease, *vpsR* transcription only slightly declined (1.9-fold). The extracellular protein secretion (*eps*) system plays a vital role in *V*. *cholerae* pathogenesis and environmental survival; it is involved in exporting toxins and VPS across the outer membrane [45]. Expression of *epsE* (the third gene of the *eps* operon) was measured to denote the change of the *eps* operon when exposed to WCESP. As shown in Fig 2, a 3.8-fold of decrease was observed when WCESP was added to the growth medium. These results suggested WCESP reduces the production and secretion of VPS and major matrix proteins.

### WCESP-mediated biofilm inhibition is independent of HapR and the quorum-sensing pathway

**Fig 5:**
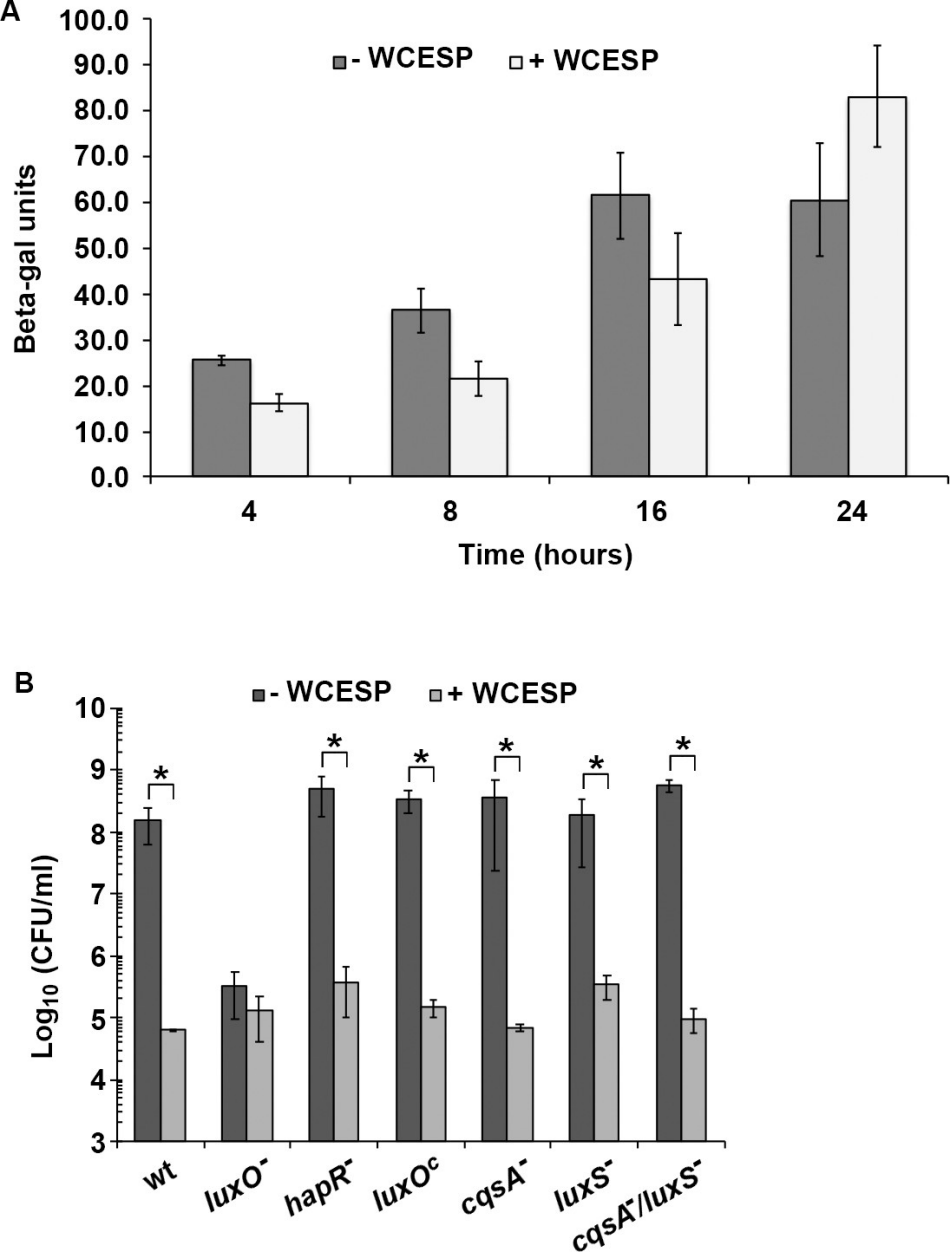
WCESP-mediated biofilm inhibition is independent of HapR and the quorum-sensing pathway. (A) β-galactosidase assay of *hapR* transcription using a *V*. *cholerae* C6706 strain carrying a chromosomal *hapR-lacZ* fusion. (B) Biofilm formation in the presence and absence of 2 mg/ml WCESP using the plate count method in *V*. *cholerae* C6706 derived mutant strains. Data shown are the average results from three independent experiments, and triplicate samples were tested each time; error bars are standard error of the mean. (* indicates *p* < 0.05).

QS pathway plays a central role in the regulation of *V*. *cholerae* biofilm, and cranberry constituents have been reported to attenuate QS-mediated cell-cell signaling in *V*. *harveyi* and *P*. *aeruginosa* [46–48]. Thus, it is natural to link WCESP’s inhibition of *V*. *cholerae* biofilm to the effects on QS system. In *V*. *cholerae*, high cell density (when CAI-1 and AI-2 concentrations are high) results in downregulation of biofilm formation through AI-sensing, LuxO dephosphorylation, and activation the master QS regulator HapR expression. HapR then inhibits *vpsR*, *vpsT*, and the *vps-*I, *vps-*II, and *rbm* genes. We reasoned that if the inhibition is through QS, then (1) WCESP would enhance the expression of HapR, and (2) in QS-deficient mutants (such as *hapR* deletion or *cqsA*, *luxS* deletions), WCESP would no longer inhibit biofilm formation. To test this, we first examined the *hapR* transcription using a strain carrying a chromosomal *hapR-lacZ* fusion [5]. As shown in Fig 5A, *hapR* transcription was slightly lower (less than 2-fold) in the presence of WCESP at T = 4, 8, and 16 hr, but at T = 24 hr, *hapR* transcription was marginally higher (1.3-fold). qRT-PCR of *hapR* from 24 hr WCESP-treated bacterial culture showed no distinct change (Fig 2). Results from the two experiments indicated WCESP does not enhance HapR expression. Next, we evaluated biofilm formation in the presence and absence of WCESP using the plate count method in the following QS-deficient mutant strains: *hapR*^*-*^, *luxO*^*c*^ (in which LuxO is locked into the active form), *cqsA*^*-*^ (no CAI-1), *luxS*^*-*^(no AI-2), and a *cqsA*^*-*^ */ luxS*^*-*^ double deletion. The *luxO*^*-*^ mutant was included to serve as a biofilm-negative control, as HapR is overexpressed in *luxO*^*-*^ and strongly inhibits biofilm formation. The results are shown in Fig 5B, in which strong inhibition of biofilm formation by WCESP was observed in all tested QS-deficient strains. Taken together, we conclude that WCESP-mediated biofilm inhibition is independent of HapR and QS pathway.

### WCESP modulates the intracellular concentration of the second messenger 3’,5’ -c-di-GMP

**Fig 6:**
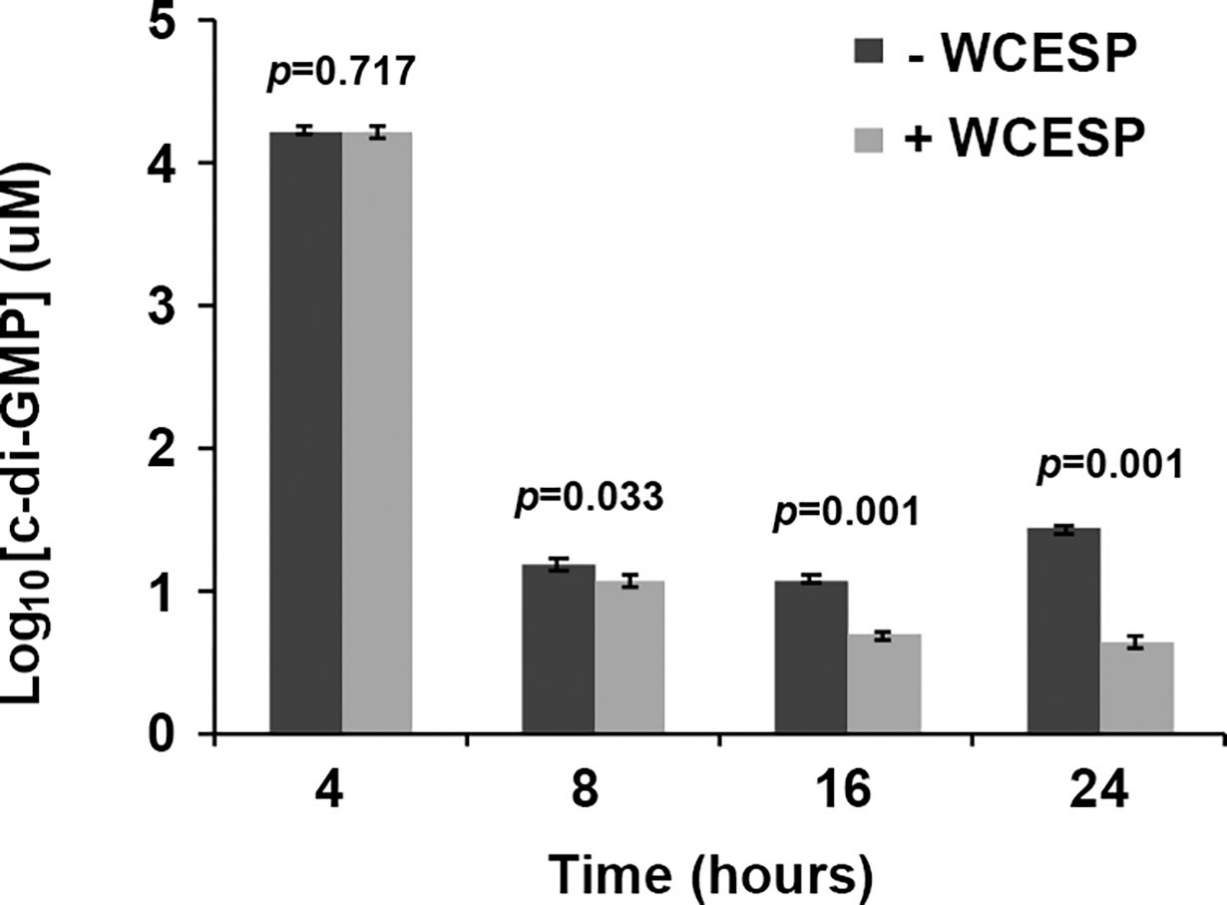
Quantification of intracellular c-di-GMP concentration in the presence and absence of 2 mg/ml WCESP using the thiazole orange (TO)-based fluorescent detection method. The concentration of c-di-GMP was calculated using a calibration curve and normalized to the concentration of a bacterial culture with 1 x 10^9^ cells/ml. Data shown are the average results from three independent experiments, and error bars are standard error of the mean.

**Fig 7:**
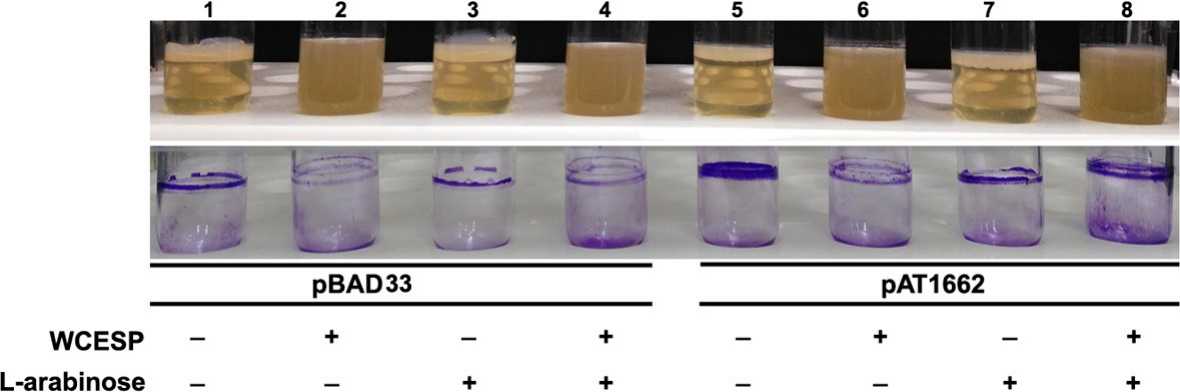
Induction of c-di-GMP synthesis partially restored biofilm formation in the presence of WCESP. The overnight bacterial culture was 1:100 diluted into 5 ml of fresh LB medium and incubated at 37°C with shaking until OD_600_ reached ~ 0.25. The culture was then aliquoted into four tubes with 1 ml each. WCESP and L-arabinose were added to the corresponding tubes with a final concentration of 2 mg/ml and 0.2%, respectively. All the tubes were subsequently incubated at 37°C for 24 hours without shaking. Tube 1–4, VC6706 with the empty vector pBAD33 to serve as controls. Tube 5–8, VC6706 with pAT1662 (pBAD33::VCA0956-His_6_).

The bacterial second messenger 3’, 5’—cyclic diguanylate (c-di-GMP) is another vital signaling system in *V*. *cholerae* that regulates the planktonic to biofilm transition and the biofilm matrix production. To examine WCESP’s influence on c-di-GMP, we sought to directly measure the intracellular c-di-GMP concentration using the thiazole orange (TO)-based fluorescent detection method [40]. To do this, *V*. *cholerae* C6706 was grown statically at 25°C in the presence and absence of 2 mg/ml WCESP. The planktonic portion of the static cultures was collected at 4, 8, 16, and 24 hours, respectively. As the bacterial cell number changes during the growth phase and also is affected by the presence of WCESP, we normalized the final c-di-GMP concentration to 1 x 10^9^ bacterial cells in 1 ml. As shown in Fig 6, no difference was found between the two 4-hr samples (which represent the initiation stage). At T = 8 hr, a slight decrease was noticeable in the WCESP-treated cells (11.8 ± 1.2 μM) as compared to the control (15.5 ± 1.5 μM). At T = 16 and 24 hr, the difference was remarkable. Results from this experiment suggest that the inhibition of *V*. *cholerae* biofilm formation by WCESP is possibly through reducing the intracellular c-di-GMP level during the development/maturation stage of the biofilm formation. If this holds true, then increasing the c-di-GMP level would at least partially counteract WCESP’s anti-biofilm effect. To test this, pAT1662 (a plasmid that contains the diguanylate cyclase VCA0956 gene under the control of the arabinose-inducible *araBAD* promoter) [32] was introduced into *V*. *cholerae* C6706 by electroporation. Biofilm formation of the resulting strain was examined when 0.2% of L-arabinose and 2 mg/ml of WCESP were added to the growth medium simultaneously. As shown in Fig 7, a partial recovery of the biofilm pellicle was observed in tube 8 (upper panel). Crystal violet staining also revealed more surface-attached cells in tube 8 (bottom panel) as compared to tube 6.

## Discussion

Due to the selective pressure of antibiotics, bacterial resistance to antibiotics is becoming an increasing health threat worldwide. A growing body of evidence demonstrated that plant extracts offer considerable antimicrobial and anti-biofilm potentials and do not come with the significant risk of antibiotic resistance. A vast number of phytochemicals have been recognized as a valuable alternative and complementary medicine to treat bacterial infections [49, 50]. The North American cranberries (*Vaccinium macrocarpon*) are rich in polyphenols, including anthocyanins, proanthocyanidins (PACs), flavonoids, and phenolic acid derivatives [51–53]. The antimicrobial activity of cranberry and cranberry products has been well acknowledged, such as preventing urinary tract disorders [54–56], dental decay [57–60], as well as stomach ulcers and cancers [61]. There are only a limited number of reports on cranberry’s anti-biofilm ability; most of these studied the effects on the periodontal pathogenic bacteria [57–59, 62–65] or the urinary tract infections (UTIs) associated pathogens [66–68]. Cranberry extracts have also been shown to reduce *Staphylococcus epidermidis* biofilm formed on soft contact lenses [69], and *Pseudomonas aeruginosa* biofilm [70]. Despite all the potential benefits, there is not enough research on cranberry’s anti-biofilm mechanism. In the present study, we investigated the effects of WCESP on *V*. *cholerae* biofilm formation. Our results indicated that sub-lethal level of WCESP could inhibit *V*. *cholerae* biofilm formation during the development/maturation stage by reducing the production and excretion of VPS and biofilm matrix proteins. We further investigated the involvement of the two key signaling systems and found that the inhibition is independent of QS and the QS master regulator HapR. On the other hand, the intracellular c-di-GMP level was decreased in the presence of WCESP. Our results imply WCESP’s anti-biofilm effect is possibly through modulating c-di-GMP signaling, though we could not rule out the possibility that other regulatory pathways might be involved.

In most bacterial pathogens, biofilm formation is activated at high cell density. Thus, inhibition of QS would result in the reduction of biofilm. But in *V*. *cholerae*, this is opposite; inhibiting QS (like the *hapR* deletion or the *luxO*^*C*^ strain) would enhance biofilm formation. Initially, we hypothesized that cranberry extract might promote QS signaling, lead to the activation of HapR, which inhibits biofilm, but our results didn’t support this idea. Meanwhile, cranberry constituents have been reported to attenuate QS-mediated cell-cell signaling in *V*. *harveyi* and *P*. *aeruginosa*. In *V*. *harveyi*, the nondialyzable material (NDM) of cranberry competes with the autoinducer for binding to its receptor [46]. In *P*. *aeruginosa*, cranberry-derived PACs (contains > 95% PACs) inhibits autoinducer production and also antagonizes the activation of QS transcriptional regulators [48]. These studies showed that the NDM or PACs is the major component in cranberry that functions as QS inhibitor. The cranberry extract used in our study is water-soluble and contain only 4% PACs; this probably explains why we did not see any impacts on QS signaling but also indicates the aqueous cranberry extract possesses anti-biofilm activity in a QS-independent mode.

The second messenger c-di-GMP has been identified in almost all major bacterial phyla, and its role as a signaling molecule involved in a variety of physiological processes has been well documented [71]. In *V*. *cholerae*, c-di-GMP controls motile to sessile transition at both the initial attachment and the matrix production/development stages. Jones et al. reported that c-di-GMP promotes the polymerization of MshA subunits to form the MshA pili which are indispensable for the initial attachment to abiotic surfaces [23]. C-di-GMP also promotes the biofilm matrix production through interaction with the two key transcriptional activators, VpsR and VpsT [24, 25]. Since the *V*. *cholerae* genome encodes numerous proteins containing the functional domains for c-di-GMP metabolism, instead of measuring the expression of individual genes, we directly measured the change of intracellular c-di-GMP concentration when WCESP is added to the growth medium. The decrease of c-di-GMP was detected in the 8, 16, and 24 hr samples, but not in the 4 hr samples. Consistent with the results from the motility, qRT-PCR, and pre-established biofilm inhibition assays, this result suggested that the soluble constituents from WCESP impede the development/maturation stage of biofilm formation through modulating the c-di-GMP level. It’s worth mentioning that we didn’t observe enhanced motility at 24 hr, when c-di-GMP level was reduced by WCESP. High concentration of c-di-GMP is known to repress *V*. *cholerea* motility [20], thus we should expect to see enhanced motility if c-di-GMP level is decreased. This discrepancy might reflect the signaling specificity among different c-di-GMP receptors or binding proteins. We propose multiple mechanisms might be involved in moderating the c-di-GMP concentration by WCESP. The active ingredients from WCESP may affect c-di-GMP synthesis and degradation through downregulating the diguanylate cyclases or upregulating the phosphodiesterases at the expression level or changing their activities; the active ingredients may also function as a c-di-GMP sequestrator to compete with the c-di-GMP receptors. In addition, WCESP may affect the communication between c-di-GMP and its receptor through binding to the receptor protein or changing its conformation or activity. Studies are underway to elucidate the molecular mechanisms. Characterization of the active ingredients from WCESP is another focus of our research.

In this study, we also tested WCESP’s anti-biofilm activity on other pathogenic bacteria, including four Gram-positive strains (*S*. *aureus*, *E*. *faecalis*, and two *L*. *monocytogenes* strains) and three Gram-negative bacteria (*P*. *aeruginosa*, *S*. *typhimurium*, and *E*. *coli O157*:*H7*). No inhibition was observed in all these strains. In contrast to *V*. *cholerae*, QS (high cell density) promotes biofilm formation in these strains. Because the content of PACs or NDM (the potential QSI) is quite low in our WCESP sample, there was probably no impact on QS signaling in these strains. In the three Gram-negative bacteria, c-di-GMP signaling has also been characterized to regulate biofilm formation, yet we didn’t see biofilm inhibition effect. Maybe higher concentrations of WCESP are required, or more importantly, each bacterium has a unique c-di-GMP signaling network.

## Conclusions

Targeting QS or the nucleotide-based second messengers is considered the new hope to address the growing problem of antibiotic resistance. While various natural and synthetic compounds have been characterized as QSIs, inhibitors of the cyclic dinucleotide signaling are still lacking. In this study, we revealed soluble constituents in cranberry extract could inhibit *V*. *cholerae* biofilm formation possibly through modulating the c-di-GMP level. Although there are more questions related to the inhibition mechanisms and the active ingredients to be answered, this work suggests that cranberry, as well as many functional foods, may contain potent inhibitors of the bacterial nucleotide-based signaling pathway and leads to a new direction of the functional food study and the antimicrobial and anti-biofilm drug development.

## Supporting information

S1 Fig WCESP inhibits *V*. *cholerae* biofilm formation at 37°C. The bacterial cultures were incubated for 24, 48, and 72 hours without shaking in the absence and presence of 2 mg/ml of WCESP.
(TIF)

S2 Fig qRT-PCR analysis of *V*. *cholerae flaA*, *pomB*, and *mshA* genes in response to 2 mg/ml WCESP (normalized to 16S rRNA) after 4 hours. Results are the average of three independent experiments, and error bars are standard error of the mean.
(TIF)

S1 Table Total *V*. *cholerae* cell count (both planktonic and biofilm cells) in the presence or absence of 2 mg/ml of WCESP during the 24-hour time course. The same experiment was repeated three times. The data shown are the representative results from one of the experiments.
(DOCX)
