# Supplementary figures and images for: Water-soluble cranberry extract inhibits Vibrio cholerae biofilm formation possibly through modulating the second messenger 3’, 5’ - Cyclic diguanylate level
Source: PLoS One. 2018 Nov 7;13(11):e0207056. doi: 10.1371/journal.pone.0207056 (PMC6221352; doi:10.1371/journal.pone.0207056)

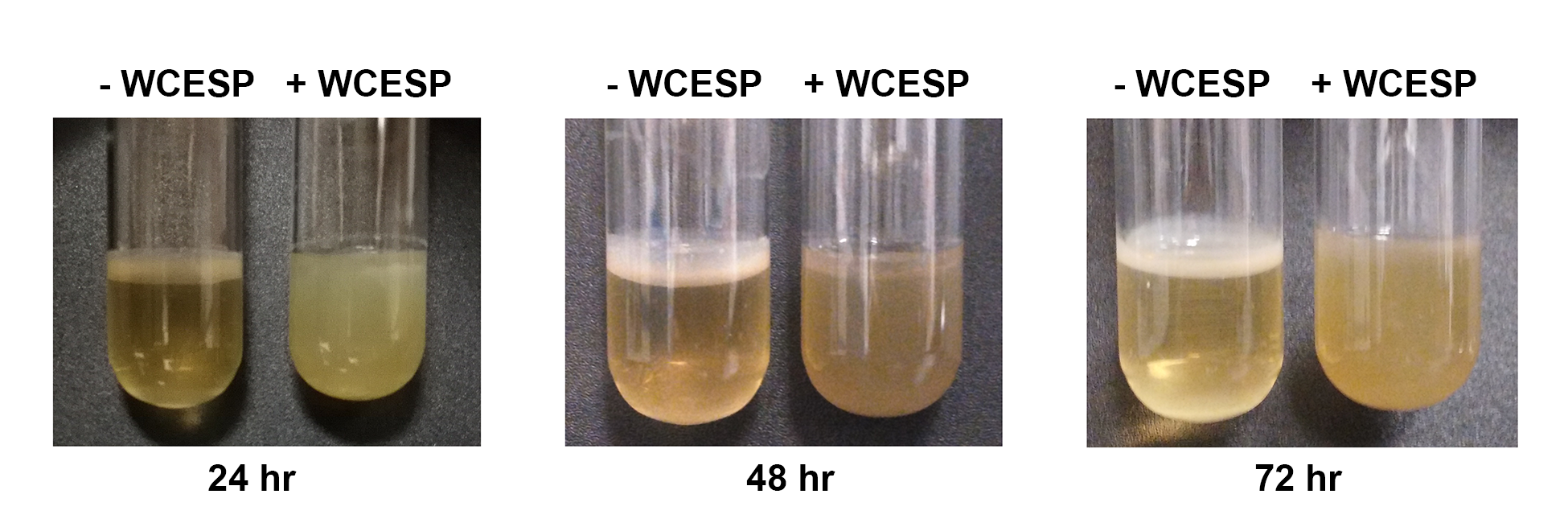

Supplement: S1 Fig — The bacterial cultures were incubated for 24, 48, and 72 hours without shaking in the absence and presence of 2 mg/ml of WCESP. (TIF) [file pone.0207056.s001.tif]

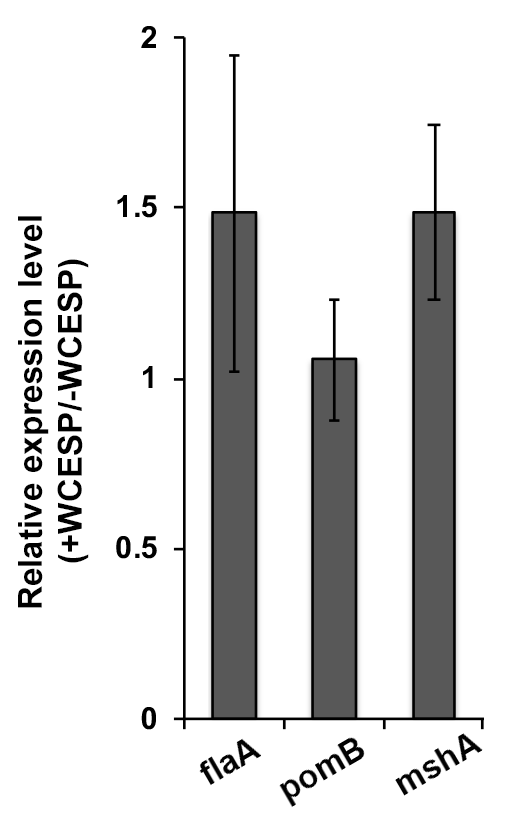

Supplement: S2 Fig — Results are the average of three independent experiments, and error bars are standard error of the mean. (TIF) [file pone.0207056.s002.tif]
