# Supplementary material for: Water-soluble cranberry extract inhibits Vibrio cholerae biofilm formation possibly through modulating the second messenger 3’, 5’ - Cyclic diguanylate level
Source: PLoS One. 2018 Nov 7;13(11):e0207056. doi: 10.1371/journal.pone.0207056 (PMC6221352; doi:10.1371/journal.pone.0207056)
Supplement: S1 Table — The same experiment was repeated three times. The data shown are the representative results from one of the experiments. (DOCX) [file pone.0207056.s003.docx]

| Incubation time (hour) | CFU’s /ml | |
| --- | --- | --- |
|  | - WCESP | + WCESP |
| 0 | 4.38 x 10^5^ | 2.12 x 10^5^ |
| 1 | 2.40 x 10^5^ | 7.40 x 10^4^ |
| 2 | 6.40 x 10^4^ | 4.88 x 10^4^ |
| 3 | 5.74 x 10^4^ | 4.90 x 10^4^ |
| 4 | 6.80 x 10^4^ | 4.58 x 10^4^ |
| 5 | 2.66 x 10^4^ | 2.86 x 10^4^ |
| 6 | 2.00 x 10^4^ | 5.86 x 10^4^ |
| 7 | 6.00 x 10^5^ | 4.34 x 10^6^ |
| 8 | 7.00 x 10^6^ | 5.00 x 10^7^ |
| 9 | 2.28 x 10^7^ | 8.20 x 10^7^ |
| 10 | 6.40 x 10^7^ | 2.02 x 10^8^ |
| 11 | 1.90 x 10^8^ | 1.26 x 10^8^ |
| 12 | 1.92 x 10^8^ | 1.28 x 10^8^ |
| 13 | 1.70 x 10^8^ | 2.08 x 10^8^ |
| 14 | 1.46 x 10^8^ | 2.20 x 10^8^ |
| 15 | 1.50 x 10^8^ | 2.40 x 10^8^ |
| 16 | 1.38 x 10^8^ | 1.68 x 10^8^ |
| 17 | 1.54 x 10^8^ | 1.90 x 10^8^ |
| 18 | 1.38 x 10^8^ | 3.96 x 10^8^ |
| 19 | 1.74 x 10^8^ | 2.66 x 10^8^ |
| 20 | 1.68 x 10^8^ | 1.10 x 10^8^ |
| 21 | 1.42 x 10^8^ | 0.98 x 10^8^ |
| 22 | 1.22 x 10^8^ | 1.92 x 10^8^ |
| 23 | 1.90 x 10^8^ | 2.46 x 10^8^ |
| 24 | 2.74 x 10^8^ | 2.04 x 10^8^ |
